# Why Are Ethnic Minorities Under-Represented in US Research Studies?

**DOI:** 10.1371/journal.pmed.0030049

**Published:** 2005-12-27

**Authors:** Aziz Sheikh

## Abstract

Sheikh discusses a new study that found that the main barrier to the participation of minority ethnic people in research lies in their reduced likelihood of being invited to participate.

Most economically developed nations are now multiethnic, and, given current demographic trends, there is reason to believe that societies will continue to become more ethnically and culturally diverse. For example, the 1991 and 2001 UK censuses, which both included a mandatory question on ethnic identity, revealed that the proportion of the UK population classifying themselves as belonging to a non-white minority ethnic group increased by 53% over this 10-year period, from 3 million to 4.6 million (or 7.9% of the UK population) [1].

We have more than two decades of research highlighting ethnic inequalities for a range of long-term disorders [2], such as asthma (Table 1), but despite the policy imperative to improve health outcomes for marginalised populations, there has, unfortunately, been little progress toward this end [3,4]. Perversely, data indicate that for some conditions these health inequalities may actually be increasing.

**Table 1:**
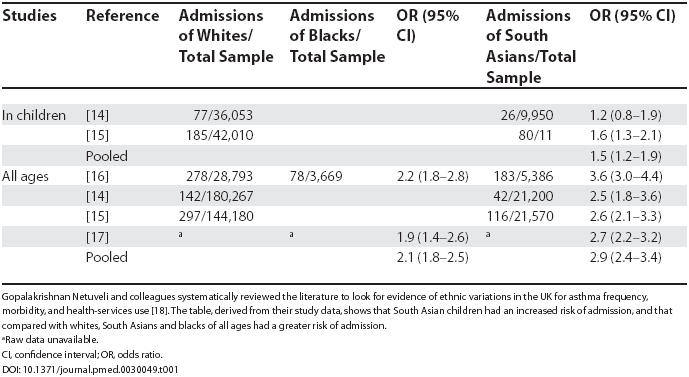
Pooled Risk of Admissions for Asthma for South Asian Children and Blacks and South Asians of All Ages, Compared with Whites

Gopalakrishnan Netuveli and colleagues systematically reviewed the literature to look for evidence of ethnic variations in the UK for asthma frequency, morbidity, and health-services use [18]. The table, derived from their study data, shows that South Asian children had an increased risk of admission, and that compared with whites, South Asians and blacks of all ages had a greater risk of admission.

^a^Raw data unavailable.

CI, confidence interval; OR, odds ratio.

Why this is the case is almost certainly dependent on an array of complex socio-economic factors [5]. Hampering efforts to reverse these trends is the lack of long-term investment into researching the health needs of minority ethnic communities and, as is increasingly being shown, evidence of their systematic under-representation in research studies in general. This lack of investment and under-representation are concerning as it may reasonably be argued that greater resources and effort should be directed toward researching those sections of society that have the greatest capacity to benefit from such research. A study in this month's *PLoS Medicine* by David Wendler and colleagues investigates one possible source of under-representation—the willingness of ethnic minorities to participate in health research [6].

## Ethnic Minorities' Willingness to Participate in Research

Previous work has shown that reporting of the ethnic profile of research participants in trials and other studies has been poor in both the US and the UK [7]. This poor reporting almost certainly reflects an underlying under-representation of these communities in these studies [8–10]. What is notable, however, is that the blame for this under-representation has typically been placed firmly at the feet of the marginalised. It is argued, often without any strong supporting evidence, that the minority ethnic groups in question either fail to understand the importance of the research process or are unable to participate because of language barriers. According to this argument, even if minority groups can comprehend the nature of the research and are able to participate, they may distrust it to the extent that they decline to participate [11,12]. Framed in such a manner, the answer to under-recruitment is seen to lie in greater integration of minority groups to the values of the majority, and the policy and research imperative is therefore to find ways of influencing the attitudes and practices of the minority communities in question.

But Wendler and colleagues' study provides strong empirical evidence to challenge the assumptions that have to date dominated discussions in this area [6]. In their systematic review, the authors set out to address the question of whether individuals from minority groups who are invited to participate in health research are less likely to consent to participate than non-minority individuals. They identified 20 health research studies that reported consent rates by race or ethnicity, 18 of which were single-site studies conducted exclusively in the US or multi-site studies where most of the sites were in the US. They found that when approached to participate, minority ethnic communities in the US are on the whole no less likely, and possibly even more likely than non-Hispanic whites, to agree to participate in research studies. Their work carefully teases out that the main barrier to the participation of ethnic minorities lies in their reduced likelihood of being invited to participate. This work thus places the burden of responsibility not on the marginalised, but on the research community: funders, ethics committees, and researchers alike.

## Strengths and Weaknesses of the Study

The key strength of this work is the rigorous systematic review methodology used to identify studies and extract and summarise data. Its main limitation relates to the failure to contact authors who collected, but did not publish, relevant data on ethnicity and consent rates. Contacting authors in this way to obtain additional data that might not be published, and also in an attempt to uncover additional unpublished material, is standard practice in most rigorously conducted systematic reviews. In addition, the fact that this work confines its focus to the US situation renders it difficult to know to what extent the findings may be generalised beyond the US experiences. As the authors point out in their discussion, the US does not guarantee universal access to health care, and perhaps individuals from ethnic minority groups may be more likely than non-Hispanic whites to use participation in research as a way to obtain access to physicians and health care.

## Next Steps

The findings from this study clearly have important and wide-ranging implications for the US research community (and possibly elsewhere as well). Funders must, for example, appreciate that to meaningfully involve ethnic minority groups in health research carries financial costs. For example, inviting individuals from these groups to participate in a study, and ensuring that they fully understand what participation involves, requires the use of interpreters and the generation of translated materials about the study—both of which are costly. Also, if sub-group analyses by ethnic groups are considered important, this will typically require considerable inflation of the sample sizes needed, thereby also increasing costs [13]. Similarly, ethics committees need to appreciate that differences in ethical values and practices across different ethnic groups need to be understood and not ignored. For example, insisting on written consent from people originating from an oral culture may unnecessarily hinder recruitment to a study; voice recordings of the consent procedure in such cases should be deemed sufficient. And as for researchers, there is a need for a better appreciation of where minority ethnic populations are located and how they are structured to allow cost-efficient recruitment and retention strategies to be developed.

There are now sufficient examples of studies on marginalised communities that clearly show that it should really be possible to engage with people, irrespective of their ethnic background, and encourage them to participate in research that is ultimately in their and/or their community's best interests. What is now needed is less blame directed at already marginalised people. Instead, those with the power to change the way in which research is conducted should translate the important insights provided by Wendler and colleagues' study into significantly more invitations extended to minority ethnic and racial groups to participate in the research endeavour.
